# Differential integrative omic analysis for mechanism insights and biomarker discovery of abnormal Savda syndrome and its unique Munziq prescription

**DOI:** 10.1038/srep27831

**Published:** 2016-06-14

**Authors:** Xia Guo, Iskandar Bakri, Abulizi Abudula, Kalbinur Arken, Mahmut Mijit, Batur Mamtimin, Halmurat Upur

**Affiliations:** 1 Shenzhen Hospital of Southern Medical University, Shenzhen 518110, PR China; 2 Xinjiang Key Laboratory of Molecular Biology and Endemic Diseases, Xinjiang Medical University, Urumqi 830011,PR China; 3 Department of Biochemistry and Molecular Biology, Xinjiang Medical University, Urumqi 830011, PR China; 4 Faculty of Traditional Uyghur Medicine, Xinjiang Medical University, Urumqi 830011, PR China; 5 Central Laboratory, Xinjiang Medical University, Urumqi 830011, PR China

## Abstract

Research has shown that many cancers have acommon pathophysiological origin and often present with similar symptoms. In terms of Traditional Uighur Medicine (TUM) *Hilit* (body fluid) theory, abnormal Savda syndrome (ASS) formed by abnormal *Hilit* is the common phenotype of complex diseases and in particular tumours. Abnormal Savda Munziq (ASMq), one representative of TUM, has been effective in the treatment of cancer since ancient times. Despite the physiopathology of ASS, the relationship between causative factors and the molecular mechanism of ASMq are not fully understood. The current study expanded upon earlier work by integrating traditional diagnostic approaches with others utilizing systems biology technology for the analysis of proteomic (iTRAQ) and metabolomic (^1^H-NMR) profiles of Uighur Medicine target organ lesion (liver) tumours. The candidate proteins were analyzed by enrichment analysis of the biological process and biomarker filters. Subsequently, 3Omics web-based tools were used to determine the relationships between proteins and appropriate metabolites. ELISA assay and IHC methods were used to verify the proteomic result; the protein von Willebrand factor (vWF) may be the “therapeutic window” of ASMq and biomarkers of ASS. This study is likely to be of great significance for the standardization and modernization of TUM.

The roots of traditional Uighur medicine(TUM) can be traced to ancient Graeco-Arab medicine and has been used to diagnose, treat and prevent illnesses for more than 3,000 years in Xinjiang, China^1^,^2^. The essence of TUM is *Hilit* (body fluids) theory. According to this theory, *Hilit* is produced by the “*liver*”, a functional organ representing multiple organs, on aninnate and acquired basis, manifesting individual body physiological and/or pathological microenvironments in many ways such as functions and metabolism in response to internal or external stimuli. It describes a disease in terms of abnormal *Hilits*, i.e. different syndromes, which are caused by disturbances of the dynamic homeostasis of Safra, Phlegm, Savda and Kan, referred to as the 4 *Hilits*. Among these, abnormal Savda is the predominant one and often develops alongside the other abnormal *Hilits*. If not prevented overtime, abnormal Savda *Hilit* can easily deposit itself against blood vessels causing dilatation, which ultimately causes the abnormal Savda syndrome (ASS), which can lead to chronic disease^1^,^2^,^3^.

ASS can be thought of as a sub-health condition, precipitated (1) in a dry, cold setting and (2) by emotional and psychological stress. Long-term studies have established that ASS may well be the underlying basis triggering chronic diseases i.e. various types of malignant tumours, etc.^4^,^5^. An epidemiological study of 3,196 complex disease cases (including cancer, hypertension, type 2 diabetes mellitus, chronic asthma, etc.) has shown unequivocally that ASS was the main common syndrome for diagnosis of TUM^6^. In particular, the proportion of ASS malignant tumours was as high as 72%, far higher than for other abnormal fluids^7^. Since ancient times, abnormal Savda-related disease treatments have centered on abnormal Savda Munziq (ASMq), which regulates the abnormal *Hilit* that causes disease, recovers the Mizaj (the temperament as paraphrase) and balances other *Hilits*^1^,^2^,^3^. Several studies found that flavonoids isolated from ASMq as an essential component can induce cell cycle arrest and cellular apoptosis of tumour cells^8^. We assumed that the pathophysiology of ASS and its related factors are associated with common mechanisms of pathogenesis and carcinogenesis, but the mechanism remain to be elucidated, which will be the key to prevention and treatment. Compared with biomolecular sciences and Western-based medicine, the TUM approach to diagnostics appears unobjective, lacking in long-standing phenotypic-rich insights and in quantitative diagnostic criteria^9^,^10^. Previously, we examined the serum proteomic profile of 29 patients with breast, lung and gastric cancer who underwent treatment of abnormal Savda with its unique ASMq prescription and found that 31 and 27 proteins were differentially expressed in ASS cancer patients and in response to treatment^5^. To verify the results of the clinical analysis, in the present study we established an experimental rat ASS hepatocarcinoma (ASS HCC) model in order to verify the previous results and to analyze ASMq related serum protein changes, in order to identify the “therapeutic window” of ASMq, which will be of great significance for standardization and modernization of TUM.

## Results

### Qualitative and quantitative analysis of serum proteins that were differentially expressed

In the present study, the identification data of iTRAQ-labeled peptides in higher-energy collisional dissociation (HCD) mode with normalized collision energy (NCE) from 28% and the median intensity for normalization were evaluated. Quantitative and qualitative analyses of the differentially expressed serum proteins following Munziq treatment in patients and the animal model were carried out using Nano-LC coupled with Q-Exactive MS.

A protein can be identified uniquely by having two unique peptides labeled with iTRAQ for quantitative analysis. A total of 404 proteins from 4,183 novel peptides corresponding to 324,650 MS/MS spectra (false discovery rate <0.01) were identified. Compared with the healthy control group, 326 (a) serum proteins in the liver cancer control group and 404 (b) serum proteins in the ASS treatment control group displayed significant differences in abundance (supplemental Tables S1 and S2). Compared to the liver cancer control group, 257 (c) distinct proteins were characterized as differentially expressed in the ASS treatment control group (supplemental Table S3). Next we found 68 in (b) and (c) commonly expressed serum proteins (Tables S2 and S3) to be considered as specific proteins for ASS HCC (supplemental Table S4). Compared with the ASS treatment control group, there were 118 proteins in the low, median and high dose ASMq group differentially expressed at the same time. Then we selected common expression protein patterns of both the 68 and 118 proteins and identified 29 (Table S5) proteins considered to be specific core proteins involved in ASMq treatment (supplemental Fig. S1).

### Characteristics of ASS animal model

Compared to the control group, the tongue texture of the ASS treatment control group was dark purple (supplemental Fig. S2A). The ASS treatment control group presented with signs including irritability, limp limbs and fell hysteretic action, most of the time like to stay together (supplemental Fig. S2B). Later gradually appeared no resistance or irritable mood after long-term stimulation, weight loss, poor sleep and bad eating. The morphological presence of a liver tumour reveals abnormality and malignant characteristics (supplemental Fig. S3A). The ASS treatment control liver cells showed granular degeneration, fibroplastic proliferation as well as the formation of cell clusters (supplemental Fig. S3B). The weight was reduced and the liver weight difference was statistically significant between the groups (*P* < 0.05) (Table 1).

### Identification of discriminatory metabolites

Representative 3D plots of the PLS-DA score in ^1^H-NMR spectra from controls vs model vs ASS model group are shown in supplemental Fig. S4A, and the ASS model vs ASMq medium dose group are shown in supplemental Fig. S4B. Abundant endogenous metabolites were detected and confirmed by using 2D NMR methods such as TOCSY, COSY and J-Res spectra in serum according to the spectral data. The dominant metabolites confirmed in serum included:the amino acids citrulline,alanine, glutamine, phenylalanine and tyrosine; a number of sugars including α-and β-glucose; and acidic metabolites such as lactate and formic acid, and other metabolites such as glycoprotein (Table 2A). Compared with the healthy group, a wide range of amino acids including serum alanine, glycoprotein, citric acid, tyrosine and phenylalanine acid content were increased while the concentration level of α-glucose and β-glucose were decreased in the other groups.

We found increased concentrations of α-glucose, β-glucose and taurine in the ASMq low and medium dose groups. Lactic acid was decreased while some essential amino acids such as leucine, valine, alanine glutamine, methylhistidine were increased in the medium dose group, but no such trend changes were found in serum of the ASMq high dose group (Table 2B).

### Networks analysis and biomarker assessment of identified proteins

**Figure 1:**
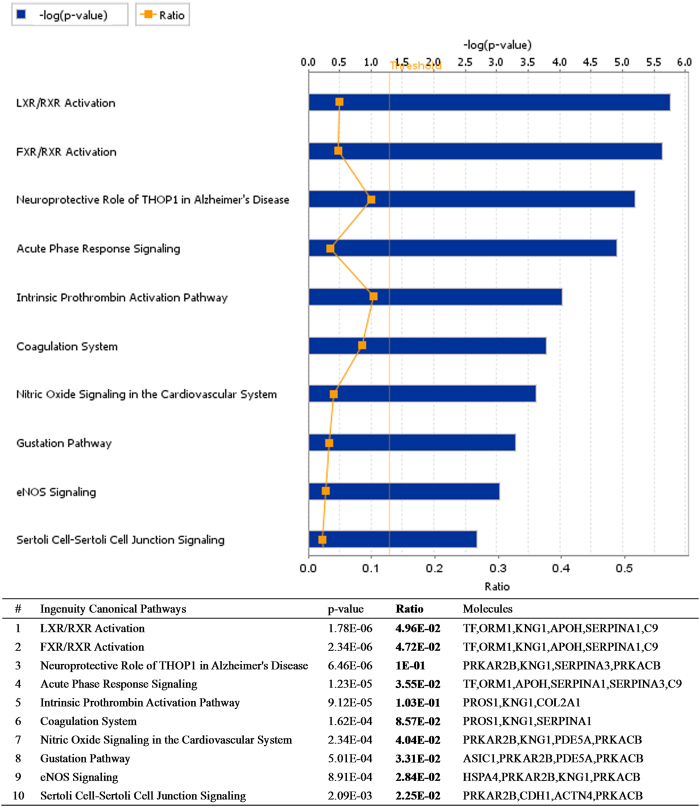
Role of 68 candidate proteins specific to ASS HCC in biological processes and molecular function determined using IPA software analysis.

**Figure 2:**
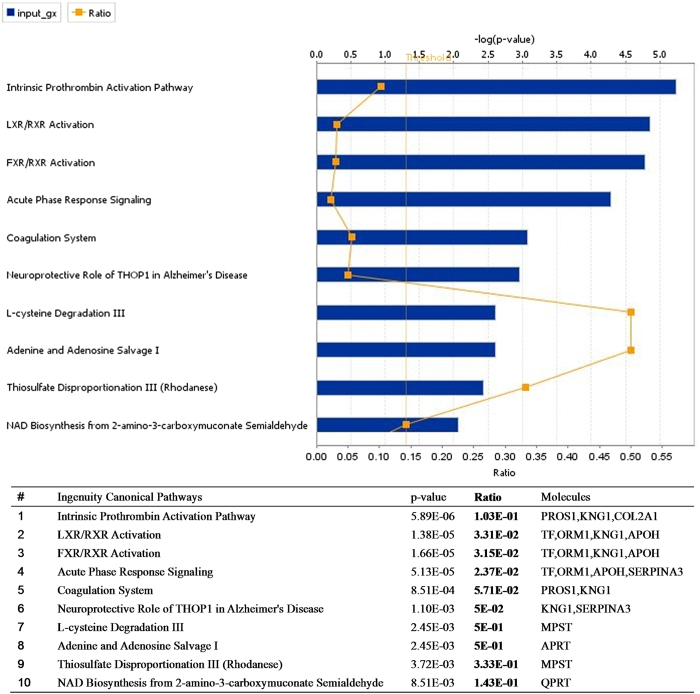
Role of 29 candidate proteins specific to ASMq in biological processes and molecular function determined using IPA software analysis.

Sixty-eight proteins specific to ASS HCC and 29 proteins specific to ASMq treatment were identified by mass spectrometry. They were analyzed by IPA^@^ core analysis in the PANTHER database according to their cellular components, biological process or molecular functions, according to Gene Ontology (GO). The 68 proteins specific to ASS HCC were placed in groups consisting of response to wounding, inflammation and hypoxia; blood coagulation and hemostasis; protein maturation by peptide bond cleavage and proteolysis, for the classification of biological processes (Fig. 1). The proteins related to ASMq were classified into groups consisting of response to wounding and healing, blood coagulation and hemostasis, inflammatory response and regulation of body fluid levels (Fig. 2). Most of the identified proteins related to ASS and ASMq were secreted proteins and transporters located in the extracellular region. When associated with canonical pathways, proteins related to ASS were divided into classes consisting of LXR/RXR Activation; FXR/RXR Activation; Acute Phase Response Signaling; Neuroprotective Role of THOP1 in Alzheimer’s Disease; the Intrinsic Prothrombin Activation Pathway and Coagulation System; Nitric Oxide and eNOS signaling (Fig. 1). The proteins related to ASMq had similar canonical pathways to ASS (Fig. 2).

Candidate biomarkers were screened from identified proteins using the expression “liver cancer” provided in GeneGo diseases ontology using the Biomarker assessment workflow tool. The ASS HCC related biomarkers (Table 3A) were mainly: metabolic enzymes (PDE5A, ALDOA, ACAA1, SERPINA1), heat shock protein (HSPA4), cadherin (CDH1), keratin (KRT1), nucleosome assembly protein (NAP1L4) and glycoprotein (ORM1). There were 2 proteins (NAP1L4 and ORM1) also in the list of ASMq treatment related biomarkers (Table 3B).

### Inter-omic correlation analysis, integration and visualization with 3Omics

It is possible to generate correlation networks automatically, with chemicals clustered according to their similar behaviour over time or in different experiments. The nodes denoted by the circles and triangles represent metabolites and proteins, respectively. Enzyme and metabolite can be represented by a correlation network. Correlated relationships (PCC > 0.9) are represented by solid lines and the text-mining results, between pairs of input molecules and other molecules, are indicated by the dotted lines. The relationships between identified proteins and metabolites related to ASS HCC in serum were analyzed by 3Omics web-based tools. The results revealed that glutamic acid (33032, 611) had a close cause-and-effect link with identified serum proteins, which could activate and be activated by them (supplemental Fig. S5A). However, supplemental Fig. S5B shows that the candidate proteins were distributed in dispersed states and surrounded the metabolites.

### Validation of ELISA assay and IHC methods

**Figure 3:**
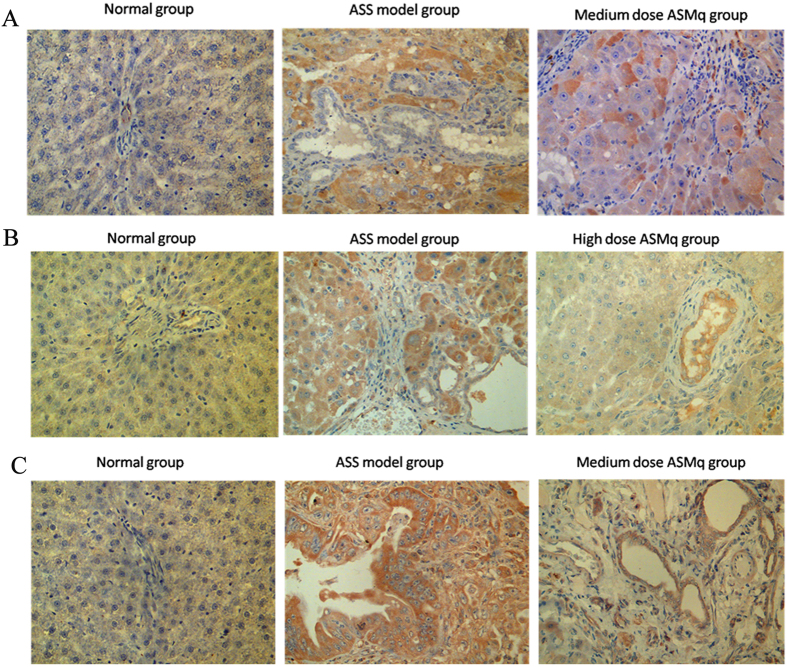
IHC analysis of the changes in vWF (**A**), SERPINA (**B**) and NAP1L4 (**C**) in healthy controls, ASS treatment controls and ASMq groups. Normal hepatic cells (healthy group) showing no immunostaining of (**A**) vWF (**B**) SERPINA and (**C**) NAP1L4 expression. Hepatocellular carcinoma cell (ASS treatment control group) showing strong (**A**) vWF (**B**) SERPINA and (**C**) NAP1L4 staining, while hepatocellular carcinoma cell (medium ASMq group) showed intermediate staining(magnification X400).

Compared to the proteomic results of our parallel experiments with cancer patients, we further verified the results of proteomics and bioinformatics analysis and detected 7 proteins that were commonly differentially expressed both in patients and in the animal model after ASS and ASMq treatment, using ELISA assay and IHC methods on serum and tissue samples. Statistical analysis showed that either in serum or in liver tissue, there was a significant upregulation of von Willebrand factor (vWF) in ASS HCC and downregulation of expression in the ASMq treatment group (*P* < 0.05) (Tables 4 and 5, Fig. 3).

## Discussion

In “*Hilit* theory” of traditional Uighur medicine, ASS is a highly pathogenic and malignant syndrome, believed to be the common pathophysiological foundation of complex disease such as tumours^11^.

For the past few years, we have studied the common biological basis of the pathological development or dysfunction of complex diseases with a high incidence in Xinjiang, e.g. malignant tumours, diabetes mellitus and bronchial asthma, based on the diagnosis of TUM by analysis of the nerve-endocrine network, pre-thrombosis state, oxidative stress and gene polymorphism, to describe such medical concepts as “same origin of different diseases” or “same treatment of similar diseases” in TUM^4^,^12^,^13^,^14^,^15^,^16^. Furthermore, we previously studied the proteomic profile of patients with different cancers (stomach, lung and breast) with ASS and demonstrated that ASS is clearly intimately involved in causal changes during carcinogenesis of the global regulation network of protein expression^5^.

However, critics question whether the diagnostic approach and remedies of Uighur medicine will meet the scientific standards necessary for international recognition, due to lack of scientific interpretations which have the same shortcomings as traditional Chinese medicine (TCM)^10^.

In order to produce unequivocal evidence of the research resultsin patients, we selected the liver as the organ in which abnormal *Hilits* are mainly produced and established an experimental hepatocarcinoma model in the rat with both ASS and ASMq treatments. We applied iTRAQ and ^1^H NMR technologies for proteomic and metabolomic analyses, to elucidate the biological essence of pathogenesis and carcinogenesis of ASS in order to interpret the molecular mechanism(s) of drug protection involved in the traditional Uighur medicine “ASMq”.

The proteomics profile using iTRAQ analysis of differentially expressed serum proteins in hepatocarcinoma model rats with ASS (ASS HCC group), ASMq treatment (low, medium, high dose), and normal controls (NC) being determined. The proteomics data revealed a total of 68 proteins specific to ASS HCC and 29 proteins specific to ASMq treatment in the ratmodel.

In the present study, the candidate proteins were analyzed using the online software IPA^®^version 8.7 and further classified according to the known biological functions of these proteins, into different intracellular compartments, types of biological processes, and canonical pathways that are associated with abnormal development of organs or disease progression. The results showed that the expressed proteins were mainly secreted proteins and transporters that were localized in the extracellular matrix or cytoplasm.

Liver, as the main target of the viscera, is generally considered to be responsible for the control of feelings and vulnerability to psychological stress-emotions pathopoiesis in TUM^1^,^11^. Additionally, the core pathway enrichment analysis of ASS related proteins revealed that some proteins function as activators that are involved in the neuro-protective role of THOP1 signaling cascades in Alzheimer’s disease. In our previous research, it was shown that a cold dry environment, diet, a variety of physical and mental stimulations, and physical or chemical stimuli can ultimately lead to ASS as a function of the intensity and duration of stimulation; this may be related to disorders of the neuroendocrine-immune system. The latter finding is also one of the important reasons why abnormal Savda *Hilit* facilitates the development of tumours. Further studies have also confirmed that the development of ASS was not only associated with an alteration of a profile specific to an organ or tissue, but also that morphological changes in multiple organs were also involved, mainly the organs belonging to the hypothalamic-pituitary-adrenal axis and the “dominant organs” (brain, liver and heart) described in TUM^14^,^17^. The core pathway enrichment analysis of ASMq related proteins found that perhaps the most important function was regulation of body fluid levels, which coincides with TUM theory. ASMq (patent no. ZL02130082.8) is a herbal formula listed in the Chinese Uyghur pharmacopoeia, and is comprised of 10 medicinal herbs. Therapy for abnormal Savda-related disease is completed by the maturation and balance the abnormal *Hilit* (fluid) with ASMq followed by the elimination of the concentrated or precipitated abnormal *Hilit* from the body with abnormal *Hilit* Mushil, which recovers the Mizaj (the temperament as paraphrase) and finally frees the body from disease. The research has shown that ASMq had anti-carcinogenic and immunomodulatory effects and so on^18^,^19^,^20^,^21^. Our study strongly suggests that the medium dose of ASMq had the strongest efficacy and had minimal toxicity.

The 6 proteins were found commonly to be differentially expressed both in patients (parallel experiment) and in the animal model specific to the ASS HCC and ASMq treatment groups, further verified using ELISA and IHC methods in serum and tissue samples taken from the animal model. Statistical analysis showed a significant upregulation of von Willebrand factor (vWF), which is a multimeric adhesive glycoprotein encoded as both an antihaemophilic factor carrier and a platelet-vessel wall mediator involved in the blood coagulation cascade^22^ in ASS HCC, which notably had downregulated expression in the ASMq treatment group (*P* < 0.05). Its plasma level increases in neoplastic diseases and arises from adverse changes in the endothelium. Hagag *et al*. found that in children with acute lymphoblastic leukaemia, there was a significant increase in soluble thrombomodulin and vWF levels during the acute phase of the disease^23^.

The results of metabolic profiling by NMR technology showed that in the serum of ASS rats with hepatocarcinoma, compared with the normal group, a wide range of amino acids levels were increased while the concentration levels of α-glucose and β-glucose were decreased. Lactic acid and citric acid levels were increased while glucose was obviously decreased compared with the control group (*P* < 0.05). Our results showed that in ASS rats with hepatocarcinoma, lipid metabolism-related metabolites are increased. The ASS may be accompanied with an imbalance of amino metabolic dysfunction of the organism, energy metabolism, increasing lipid mobilization or a decreaseing glycolysis, leading to an increased accumulation of lactic acid and the capacity of hepatic extra lipid transport, resulting in increases in the levels of serum alanine, glycoprotein, citric acid, tyrosine, phenylalanine and formic acid; however, the levels of α-glucose and β-glucose were decreased. So, it was an interesting finding which showed not only a modest Warburg shift to glycolysis but also a major upregulation of fatty acid catabolism due to metabolic reprogramming of ASS hepatocarcinoma. Perhaps ASMq regulates amino acid and glucose metabolism in the abnormal Savda hepatoma model by increasing the content of branched-chain amino acids and accelerating gluconeogenesis, in order to increase energy metabolism and supply, and reduce the accumulation of lactic acid.

In conclusion, we have demonstrated that ASS may be causally associated with changes in the whole regulatory network of protein expression during carcinogenesis. Combining proteomics and metabolomics, exquisitely and accurately distinguished the key proteins, metabolites and pathways involved, which had a function in early warning and distinguishing of ASS cancer, and be vital for monitoring the clinical treatment of drug ASMq protection. Such studies will facilitate our interpretation of the molecular mechanisms of drug protection underlying traditional Uighur medicine “ASMq”. Such studies will help to elucidate the biological essence of pathogenesis and carcinogenesis and provide a reliable theoretical basis for ASS, and to identify the “therapeutic window” of ASMq, which is of great significance for the standardization and modernization of the traditional Uighur medicine.

## Methods

### Animals and treatment

150 Wistar SPF rats (male, 4–6 weeks, 150 ± 30 g) were provided from The Laboratory Animal Centre of Xinjiang Medical University (Permit No. SYXK (Xin) 2011-0001) and kept under standard laboratory conditions, with a 12-h light, 12-h dark illumination cycle at 25 °C. The rat shad unlimited access to rodent chow and water *ad libitum*. All experimental protocols were approved by the Ethics Committee of the Medical University of Xinjiang. And the methods of this study were carried out in accordance with the relevant guidelines, including any relevant details.

### Preparation of ASMq ethanol extract

The herbal formula known as abnormal Savda Munziq is a Uighur concoction of herbs, which is used as a medication. It is composed of 10 medicinal herbs including *Adiantum capillus-veneris L., Alhagi pseudoalhagi Desv., Anchusaitalica Retz., Cordia dichotoma G. Forst., Euphorbia humifusa Willd., Foeniculum vulgare Mill., Glycyrrhizauralensis Fisch., Lavandula angustifolia Mill., Melissa officinalis L*, and *Ziziphus jujuba Mill*. (Patent No. ZL02130082.8). All plant species were confirmed by the institute for drug control (Urumqi 830002, P.R. China). In brief, the minced herbs were first rotated in 95% ethanol 1:10 (w/v) for 5 h and repeated for additional three times. The extraction of crude were filtered and distilled to oiliness form, and then degreased in petroleum ether. The extract that was water soluble and then was dried in a vacuum (Buchi, Switzerland) to produce a powder and stored at 4 °C^24^. Extract yield of plant material was 12% (w/w). In this study, the dried powder was dissolved in distilled water as a 1 g/mL stock solution and be kept at −20 °C for 1 months. Before application of ASMq, the drug must been subject to a strict quality control. Quality of the six phenolic compounds in ASMq, including gallic acid protocatechuic acid, caffeic acid, rutin, rosmarinic acid, and luteolin were assessed by reversed-phase high-performance liquid chromatography coupled with a diode array detector, as a quality control approach to ASMq^25^. The six phenolic compounds were separated on an Agilent TC-C18 reversed-phase analytical column (4.6 × 250 mm, 5 μm) by gradient elution using 0.3% aqueous formic acid (v/v) and 0.3% methanol formic acid (v/v) at 1.0 mL/min.

### Treatments

The animals were divided at random into abnormal Savda liver cancer (*n* = 100) and control (*n* = 50) groups. The control group rats were raised at 25 ± 3 °C with a relative humidity between 60 ~ 80% and fed with normal chow for 21 days. Then, they were further subdivided into a liver cancer control group (*n* = 25), which received 1 mg/mL diethylnitrosamine (DEN) in their drinking water per day and a healthy control group (*n* = 25) that received normal saline (20 μL/kg) per day for 20 weeks, with the same environmental conditions as previouslydescribed.

The 100 abnormal Savda liver cancer animals were raised for 21 days in a dry and cold feeding environment of 6 ± 1 °C with a relative humidity between 25% and 32.8% (Shanghai Jinhong Electromechanical Equipment Factory, China). The rats were fed with dry and cold food (the ratio of normal food, barley and coriander seeds in dry and cold food was 7:1.5:1.5). At the same time, intermittent plantar electric stimulations (first week 25 min, 20 V, once a day; second week, 30 min, 25 V, once a day; third week, 35 min, 30 V, once a day) were conducted using a small animal apparatus designed to deliver foot shocks (Shanghai Jinhong Electromechanical Equipment Factory, China) and immobilization periods (first week, 40 min, once a day; second week, 60 min, once a day; third week, 90 min, once a day) were imposed. In addition, the rats were forced to swim in cold water (20 ± 5 °C) once a day for 5 min. The multiple stimuli conducted for 21 days successfully established the abnormal Savda model. After 21 days ASS stress treatment, the rats were permitted to continuously drink DEN.

(1 mg/mL) for tumour induction for 20 weeks in which the animals were kept in a cold, dry environment (6 ± 1 °C), with the relative humidity between 11:00 am to 9:00 pm set at 25–32.8%. As a result, the rats suffered from induced-stress after they were trained with repeated electric foot-shocks everyday (20–30 V at intervals of 0.2–0.5 s for 20 min). At the start of tumour induction, the 100 abnormal Savda liver cancer animals were further divided into a treatment control group (*n* = 25) without any medication, a low dose ASMq group (1 g/mL ASMq daily gavage 0.6 mL, *n* = 25), a medium dose ASMq group (1 g/mL ASMq daily gavage 1.2 mL , *n* = 25) and a high dose ASMq group with 2 g/mL ASMq applied gavage 1.2 mL per day.

After stress treatment for 20 weeks, following intraperitoneal injection of anesthesiaaliquots of blood were drawn from the abdominal aorta into BD Vacutainer tubes (no additives), which were then centrifuged at 3,000 rpm (4 °C, 5 min). The serum was stored as 250 μL aliquots at −80 °C until required for measurements. The tissue of liver lesions were stored in a jar of formaldehyde and processed for routine histopathology and hematoxylin and eosin (H&E) staining. A pathologist examined the tumour slices that had been stained with H&E and classified themaccording to well-established criteria.

### Enrichment of low-abundance proteins

For proteomics analysis, serum samples from 5 rat models in each group were mixed in equal volume to form pooled samples. We used a prepacked affinity LC column (1 mL) for depleting the most common proteins by ProteoMiner^TM^ Protein Enrichment Kit (Bio-Rad), because the pooled serum samples were enriched with low-abundance proteins; protein depletion was performed according to the manufacturer’s recommended protocol. After enrichment, the protein content of each group was determined using the Bradford method. Moreover, the protein integrity of each group was detected by SDS-PAGE.

### iTRAQ labelingand in solution digestion

The methods for the iTRAQ/Shotgun experiments were performed according to the manufacturers guidelines. For protein digestion, 1.65 μL of trypsin (1 μg/uL) was added into a protein solution (50 μg) at 37 °C for 24 h. Subsequently, 1 μL of trypsin (1 μg/μL) was added for sample digestion for a further 12 hours at 37 °C. The precipitates were dissolved in 0.5 M TEAB (30 mL) and then isopropanol (70 μL)added. The digested protein samples acquired from control, liver cancer control, treatment control and ASS low, medium and high dose ASMq groups were labeled with iTRAQ^®^ Reagent-8 Plex Multiplex Kit (Applied Biosystems, MA, USA) 113, 114, 115, 116 and 117, respectively. Labeling was conducted and subsequently the peptides were combined into one sample.

### Labeled peptides by using RP nanoLC-MS/MS and SCX analysis

The desiccated combined peptides (Speedvac) were dissolved in 1 mL of buffer A (10 mM KH_2_PO_4_ in 25% ACN, pH 3.0). If required, each sample was adjusted to pH 3.0 with H_3_PO_4_. The samples were then ran through a HPLC system (Shimadzu, Kyoto, Japan) to fractionate them; a silica-based Luna SCX column 250 mm × 64.6 mm (Phenomenex, Torrance, CA, USA) was used with a strong cation-exchange (SCX) column. Thirty-eight fractions were collected using buffer B (10 mM KH_2_PO_4_, 2 M KCL in 25% ACN, pH 3.0) with the following gradient: 0%, 35 min; 0–5%, 1 min; 5–30%, 20 min; 30–50%, 5 min; 50%, 5 min, 50–100%, 5 min and 100% for 10 min. Fractions were desalted following the manufacturer’s protocol of strata-X C18 (Phenomenex) and dried with a Speedvac. Each SCX fraction was runin triplicate on a HPLC system (Shimadzu, Kyoto, Japan), and fractionated for 65 min in buffer A (H_2_O and 0.1% formic acid) and buffer B (acetonitrile and 0.1% formic acid) at a flow rate of 400 nL/min in a Q-Exactive mass spectrometer (Thermo Fisher Scientific, MA, USA). A 10 cm reversed phase C18 column (Agela Technologies; ID 75 mm, 5 μm particles, 300 A° aperture, 15 cm) was used. Full scans (positive mode) were carried out within an m/z range of 350–70,000 Da. Data-dependent tandem mass spectrometry scans (MS/MS) were ranat a collision energy of 28% and a capillary temperature of 320 °C in a high energy collisional dissociation (HCD) mode. The electrospray voltage was 1.8 kV with a resolution ratio of 17,500.

### Data analysis and protein identification method

Proteome Discoverer 1.3 (Thermo Fisher Scientific, Waltham, MA) was used for the identification of iTRAQ/Shot gun proteins with the MGF format converted from the MS/MS raw data. The MGF files were then interrogated by an in-house Mascot 2.3 (Matrix Science, Boston, MA) and compared to database uniprot 2014_rattus (building date: 2014.3.3; number of sequences: 7,889). The parameters in Mascot were optimized to identify peptides; the searchincluded: carbamidomethylation for cysteine as a set modification, oxidation of methionine, iTRAQ 8-plex modification of Gln-Pyro-Glu of the N terminus, and K for quantification andthe acceptance of one trypsin missed cleavage. The precursor tolerance for each peptide was 15 ppm and the MS/MS tolerance was 20 mmu.

### ^1^H-NMR spectroscopy of serum samples obtained from the animal model

Serum samples from different animal models were examined by using the ^1^H-Nuclear Magnetic Resonance (^1^H-NMR) method at a frequency of 500.13 MHz with a Bruker 500 NMR scanner (Rheinstetten, Germany) being employed at 298 K in accordance with previously established methods^26^. Several 2D NMR experiments were applied to select samples for assignment purposes, including ^1^H-^1^H homonuclear correlation spectroscopy (COSY), total correlation spectroscopy (TOCSY) and J-resolved spectroscopy (J-Res). For COSY and TOCSY experiments, the spectral width was selected as 10 ppm in both dimensions; the spectra resulted from 48 transients per increment and 256 increments were converted into 2048 data points. For J-Res experiments, there were 32 transients per increment and 256 increments were converted into 2048 data points with the spectral width were 10 ppm of X dimension and 1.0 ppm of Y dimension^27^.

Serum samples to be analyzed by NMR were prepared by adding 200 μL serum to 400 μL saline buffer solution (0.045 M NaH_2_PO_4_ + 0.045M K_2_HPO_4_ in 20% v/v D_2_O and 80% v/v H_2_O, pH7.4). The supernatant (550 μL) was stored at room temperature for 10 min, and then centrifuged at 10,000 × g for 10 min. The supernatant (550 μL) was aspirated and placed in a 5 mm NMR tube for NMR analysis. The ^1^H-NMR spectrum of each sample was recorded using a Carr-Purcell-Meboom-Gillpulse sequence (relaxation delay −90° − (τ − 180° − τ) n-acquire) at a proton frequency of 599.95 MHz. Sixty-four scans were converted into 32,768 data points for each sample with a spectral width of 20 ppm; the acquisition time was 1.64 s and there laxation delay was 2 s at 298 K.

### Spectral processing and metabolite identification

TopSpin 2.1 (Bruker BioSpin, Rheinstetten, Germany) was used to process NMR spectra including Fourier transform, phasing and baseline correction. Normalized NMR data sets were used to carry out pattern recognition analysis with the SIMCA-P+ software (Version 11.0, Umetrics Inc., Umeå, Sweden). Major component analysis and orthogonal projections to latent structures with discriminant analysis (OPLS-DA) methods with unit variance (UV) scaling were used to identify biomarkers and for class discrimination.

In the present study, OPLS-DA data from NMR spectra obtained from healthy control, liver cancer control, treatment control as well as low, medium and high dose ASS groups were compared. A comparison of date obtained from NMR spectra from different groups was also performed. An OPLS-DA model was developed with the NMR data as the X matrix and the class information identifier for different groups as the Y variables, using one PLS and one orthogonal factor^26^,^27^. The reliability of the OPLS-DA model was determined by the parameters R^2^X and Q^2^. R^2^X represents the total variation of the X matrix and Q^2^ the statistical predictability of the model. OPLS-DA coefficient plots were generated with MATLAB (Version 7.0) scripts. A coefficient plot demonstrated that the variables contributed to classification and its significance.

### Ingenuity pathway analysis of candidate proteins

Each differentially expressed protein was characterized by Biomarker^®^ Filter analysis and core analysis using IPA^®^software package (Version 8.7) to gain insight into the molecular mechanism of each protein and its possible potential role as a biomarker.

### Inter-omic correlation analysis, integration and visualization with 3Omics

3Omics is an online software program that generates inter-omic correlation networks to facilitate the visualization of relationships in data, with a particular emphasis on establishing links between proteins and metabolites^28^. We selected the Proteomics-Metabolomics (P-M) as our analysis method and uploaded the required input data; T and P data from NMR and iTRAQ analysis. 3Omics software offers coexpression, correlation, phenotype, GO enrichment, and pathway enrichment analyses. The “corr” function from R is used to calculate the Pearson correlation coefficient (PCC). To generate visualizations, the software uses a force-directed layout algorithm. Repulsion and attraction parameters and the correlation coefficient threshold can be finely tuned to achieve improved visualization; the default settings were 0.9, 160 and 80, respectively.

### Confirmatory experiments with ELISA and IHC

In our parallel study, we examined the serum proteomic profile of 29 cancer patients (breast, lung and gastric cancer) who underwent treatment of abnormal Savda with its unique ASMq prescription and found that 31 and 27 proteins, respectively were differentially expressed in ASS cancer patients in response to the treatment. To verify the results of proteomics and bioinformatics analysis, we selected the common differentially expressed proteins both in patients and in the ASS and ASMq treatment animal model. All serum and liver lesion tissue samples of animal models were analyzed using ELISA (USCN Life Science Inc.,Wuhan, China) and an immunohistochemical method (IHC) according to the manufacturer’s protocols. Final data from ELISA analysis was confirmed by 3 independent recognitions of each serum protein and 2 pathologists independently examined the immunohistochemistry slides. The pathologistsused an immunohistochemical scoring system (IHS) based on the German immunoreactive score^29^. The raw data were converted to IHS by multiplying the staining intensity and quantity scores together.

### Statistical analysis

The quantitative data file generated by MASCOT originating from MS/MS intensities of reporter tags, were analyzed using Scaffold Q+ (Proteome Software, Portland) with a Mann-Whitney test. For multiple comparisons between the 4 groups, an identified protein gained 4 tag intensity ratios with the corresponding *P* values when 2 parallel pairs of samples were equated. Protein abundance fold changes were calculated as the median ratio of all matched spectra with appropriate tag signals. A differential protein was required to closely match the following criteria: the fold change ratio was >1.5 or <0.8 and *P* < 0.05. Statistical analysis was carried out using SPSS version 17.0 for Windows (SPSS Inc, Chicago, Illinois, USA). The data derived from the weight growth or the determination of protein expression levels from ELISA experiments were compared using two-way ANOVA or a paired sample *t*-test. The *P* values of IHC results were calculated using the Fisher exact method.

## Additional Information

**How to cite this article**: Guo, X. *et al*. Differential integrative omic analysis for mechanism insights and biomarker discovery of abnormal Savda syndrome and its unique Munziq prescription. *Sci. Rep.* **6**, 27831; doi: 10.1038/srep27831 (2016).

## Supplementary Material

Supplementary Information

Supplementary Tables

## Figures and Tables

**Table 1:** Changes in body and liver weight after 20 weeks of DEN application.

Group	Weight (g)	Liver weight (g)
Healthy group	406.61 ± 18.19	12.25 ± 1.27
ASS model group	287.08 ± 15.52**	30.7 ± 11.62*
Liver cancer control group	310.13 ± 31.16*	18.95 ± 5.63

vs healthy group, *P < 0.05, **P < 0.01.

**Table 2:** Metabolites primarily responsible for separation in the PLS-DA Model.

Between control group and ASS model group							
No.	Metabolite	Chemical shift (ppm)	Assignment	Coefficient (r)			
					Healthy group vs liver cancer control group	Liver cancer control vs ASS treatment control group	ASS treatment control vs ASS treatment group
				R^2^X	0.77	0.62	0.68
				Q^2^	0.37	0.71	0.65
1	lactate	1.32 (d), 4.11 (q)	CH_3_, CH		0.63		
2	alanine	1.47 (d), 3.76 (q)	CH_3_, α-CH			0.72	
3	glycoprotein	2.03 (s)	NHCO-CH_3_			0.82	
4	glutamine	2.13 (m), 3.75 (t)	β-CH_2_, α-CH			−0.79	
5	citric acid	2.52 (d), 2.67 (d)	CH_2_, CH_2_			0.71	0.64
6	citrulline	1.87 (m)	β-CH2			0.80	
7	tyrosine	7.18 (d)	H_2_/H_6_			0.85	
8	phenylalanine	7.31 (m), 7.37 (m), 7.41 (m)	H_2_/H_6_, H_4_, H_3_/H_5_		0.65	0.78	
9	α-glucose	3.53 (dd), 3.72 (dd), 5.23 (d)	C-H_2_, halfCH_2_-CH_6_, C-H_1_		−0.69	−0.85	−0.68
10	β-glucose	3.40 (t), 3.89 (dd), 4.64 (d)	C-H_4_, halfCH_2_-CH_6_, C-H_1_		−0.75	−0.88	
11	formic acid	8.44 (s)	CH		0.77	−0.71	
Comparisons between the control group and the ASS model group							
No.	Metabolite	Chemical shift (ppm)	Assignment		Coefficient (r)		
					ASS treatment control vs low dose ASMq group	ASS treatment control vs medium dose ASMq group	ASS treatment control vs high dose ASMq group
				R^2^ X	0.66	0.66	0.69
				Q^2^	0.49	0.49	0.42
1	leucine	0.95 (d), 0.97 (d), 1.72 (m)	δ-CH_3_, δ-CH_3_, β-CH_2_, CH			0.68	
2	valine	1.03 (d)	CH_3_			0.69	
3	lactate	1.32 (d), 4.11 (q)	CH_3_, CH				
4	Alanine	1.47 (d), 3.76 (q)	CH_3_, α-CH			0.87	
5	glycoprotein	2.03 (s)	NHCO-CH_3_				−0.84
6	glutamine	2.13 (m), 3.75 (t)	β-CH_2_, α-CH			0.88	
7	creatine	3.03 (s), 3.93 (s)	CH_3_, CH_2_				−0.75
9	methylhistidine	7.05 (s), 7.78 (s)	H_4_, H_2_			0.68	
10	α-glucose	3.53 (dd), 3.72 (dd), 5.23 (d)	C-H_2_, halfCH_2_-CH_6_, C-H_1_		0.84	0.83	
11	β-glucose	3.40 (t), 3.89 (dd),4.64 (d)	C-H_4_, halfCH_2_-CH_6_, C-H_1_		0.83	0.83	0.74
12	taurine	3.25 (t)	CH_2_NH		0.71	0.72	0.69

Note; s is singlet; d is doublet; t is triplet;q is quartet; m is multiplet; dd is doublet of doublets.

**Table 3:** Potential biomarkers identified using IPA biomarker filter analysis.

ASS HCC related potential biomarkers				
ID	Description	Symbol	Log(Ratio)	Location
O54735	cGMP-specific 3′, 5′-cyclic phosphodiesterase	PDE5A	1.393	Cytoplasm
P68511	14-3-3 proteineta	YWHAH	1.315	Cytoplasm
O88600	Heat shock 70 kDa protein 4	HSPA4	−1.218	Cytoplasm
P05065	Fructose-bisphosphate aldolase A	ALDOA	−1.229	Cytoplasm
Q9R0T4	Cadherin-1	CDH1	−1.286	Plasma Membrane
P07871	3-ketoacyl-CoA thiolase B, peroxisomal	ACAA1	−1.326	Cytoplasm
Q6IMF3	Keratin, type II cytoskeletal 1	KRT1	−1.364	Cytoplasm
Q5U2Z3	Nucleosome assembly protein 1-like 4	NAP1L4	−1.571	Cytoplasm
P17475	Alpha-1-antiproteinase	SERPINA1	−1.784	Extracellular
P02764	Alpha-1-acid glycoprotein	ORM1	−2.789	Extracellular
B. ASMq related potential biomarkers				
ID	Description	Symbol	Log(Ratio)	Location
P02764	Alpha-1-acid glycoprotein	ORM1	−2.789	Extracellular
Q5U2Z3	Nucleosome assembly protein 1-like 4	NAP1L4	−1.571	Cytoplasm
P08934	Kininogen 1	KNG1	1.328	Extracellular space
P12346	Transferrin	TF	−1.957	Extracellular space
Q99P74	RAB27B, member RAS oncogene family	RAB27B	1.405	Cytoplasm

**Table 4:** ELISA verification of candidate protein expression in an animal model.

Serum protein level (ng/mL)				
Group	NAP1L4	KRT1	THRb	vWF
Normal	1.090 ± 0.676	2.134 ± 0.382	50.992 ± 22.094	96448.44 ± 34715.095
Model group	1.323 ± 1.525	2.034 ± 0.450	59.571 ± 22.082	161934.12 ± 38466.522^a^
ASS model group	1.693 ± 1.235	2.336 ± 0.186	50.951 ± 10.375	158014.16 ± 26783.644^a^
Low dose ASMq group	4.412 ± 4.280	1.662 ± 0.205^b^	74.069 ± 17.968^b^	152632.52 ± 26517.165
Medium dose ASMq group	2.252 ± 1.892	2.129 ± 0.267	46.617 ± 22.842	142002.12 ± 34628.528
High dose ASMq group	2.148 ± 2.955	2.106 ± 0.335	56.438 ± 24.070	150088.82 ± 52255.158
F-value	1.843	4.142	2.734	6.765
*P*-value	0.102	0.001	0.020	0.000

^a^vs normal group, *P* < 0.05; ^b^vs ASS model group, *P* < 0.05.

**Table 5:** IHC verification of candidate protein expression in liver tissue of an animal model.

Protein levelsin liver tissue				
Group	PF4	NAP1L4	SERPINA	vWF
Normal	29.1818 ± 12.600	21.8182 ± 11.7287	27.3636 ± 4.7609	32.3636 ± 14.9684
Model group	34.5385 ± 14.8358	37.6154 ± 14.4484	35.0714 ± 4.4283	54.1538 ± 12.4287
ASS model group	39.8750 ± 10.5866	33.7500 ± 14.4484	33.5000 ± 6.4807	56.2500 ± 16.2282^a^
Low dose ASMq group	39.5833 ± 9.1697	38.3077 ± 9.9614	38.3846 ± 10.8438	57.0000 ± 13.3479
Medium dose ASMq group	37.0796 ± 10.5866	26.3846 ± 13.7085	35.6923 ± 3.6602	47.3846 ± 14.5002^b^
High dose ASMq group	33.0000 ± 11.4367	32.7143 ± 3.4016	32.6667 ± 4.7609	64.5714 ± 7.9132
F value	3.296	2.962	10.036	19.146
P value	0.006	0.012	0.000	0

^a^s normal group, *P* < 0.05; ^b^vs ASS model group, *P* < 0.05.
