# Supplementary material for: Differential integrative omic analysis for mechanism insights and biomarker discovery of abnormal Savda syndrome and its unique Munziq prescription
Source: Sci Rep. 2016 Jun 14;6:27831. doi: 10.1038/srep27831 (PMC4906522; doi:10.1038/srep27831)
Supplement: Supplementary Information [file srep27831-s1.pdf]

Differential integrative omic analysis for mechanism insights and biomarker discovery of abnormal Savdasyndrome and its unique Munziq prescription

Xia Guo[1], Iskandar BAKRI[2], Abulizi Abudula[2], Kalbinur Arken[3], Mahmut Mijit[3], Batur Mamtimin[4], Halmurat Upur[5*]

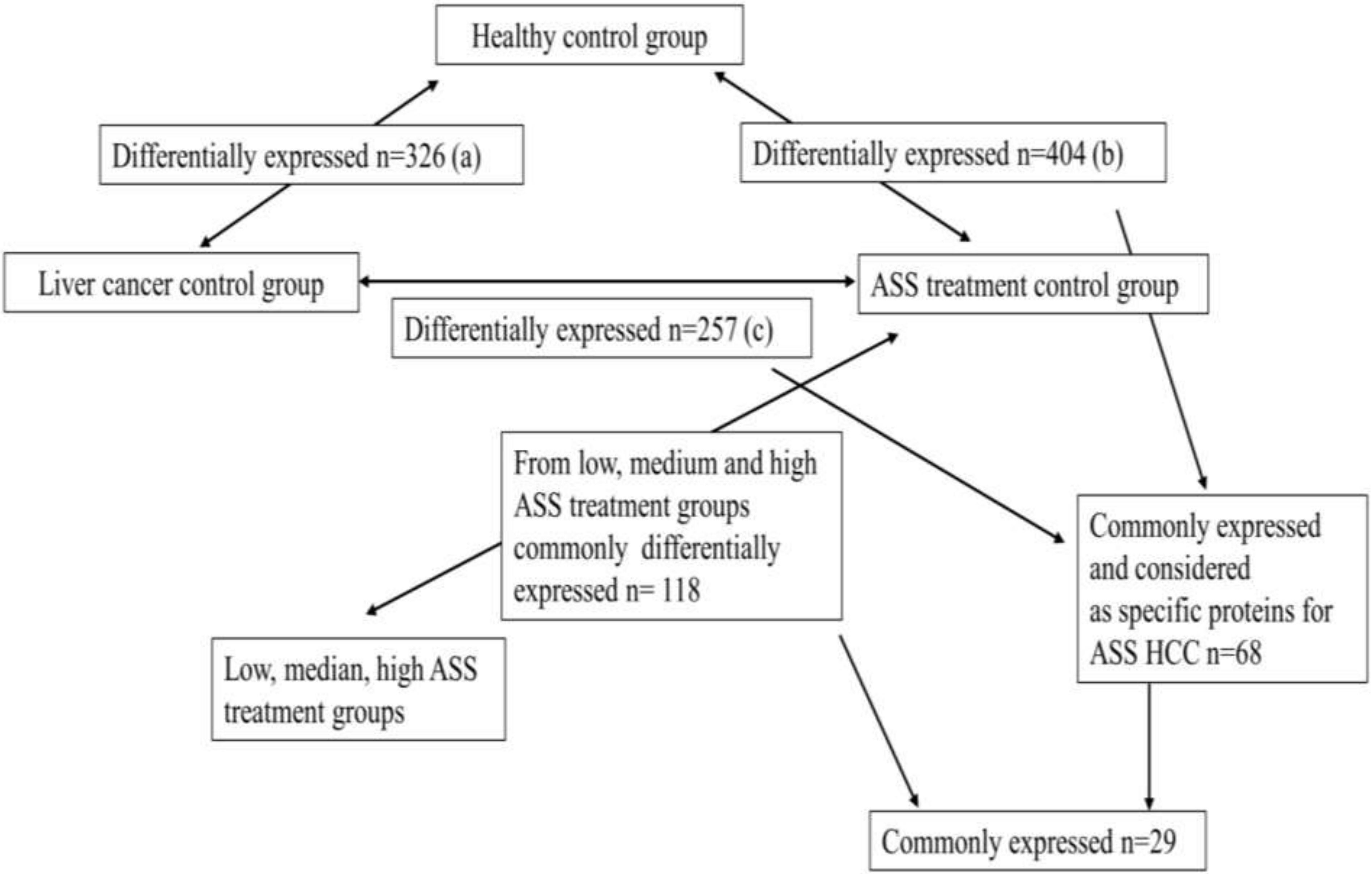

Figure S1.The scheme of the key findings of serum protein expression patterns.

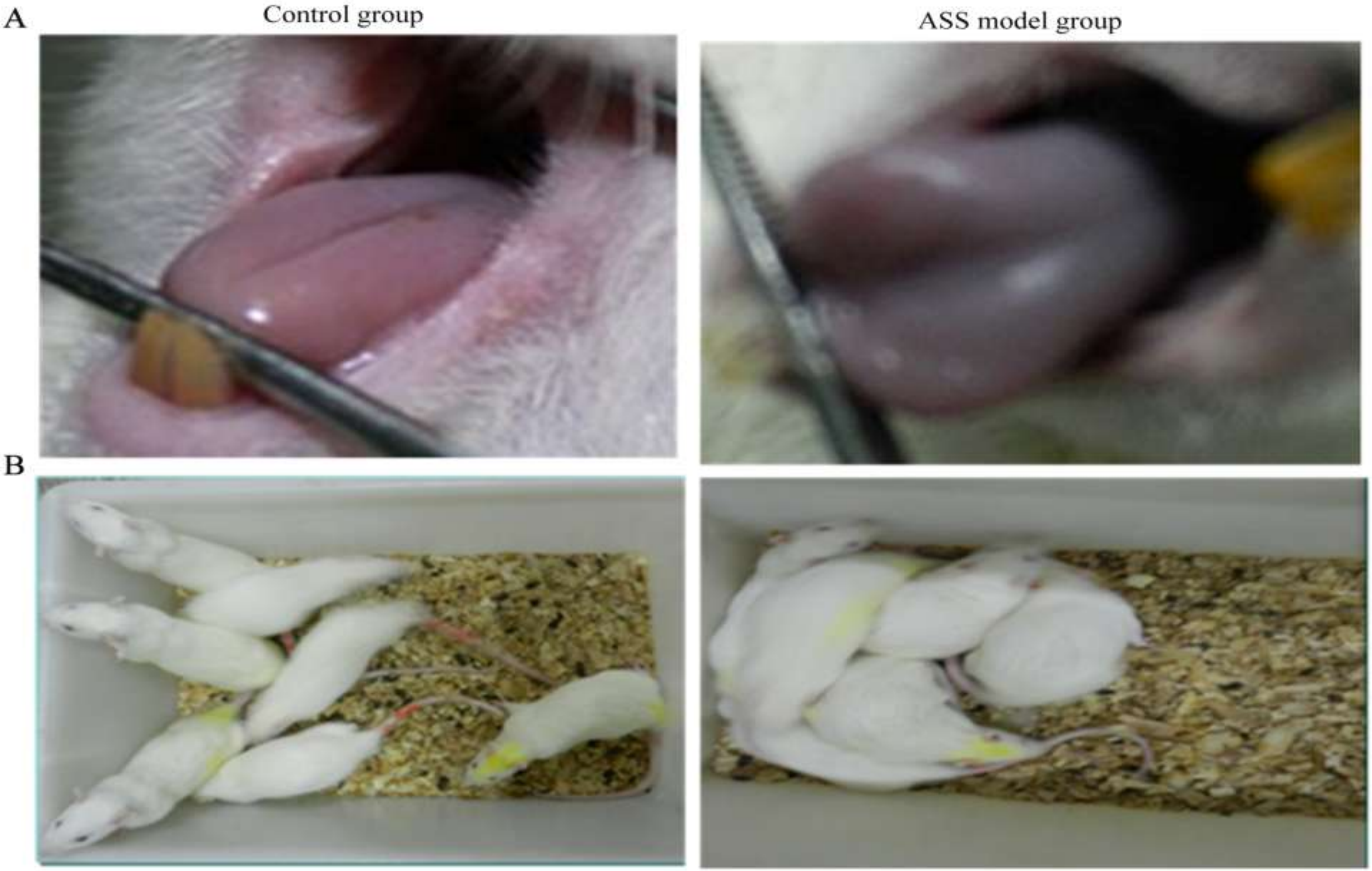

Figure S2. Changes in tongue and emotional reactions of experimental rats at the 20th week. （A) The control had a normal tongue where as the tongues of the ASS treatment control rats were dark purple. （B) Control rats moved freely, whereas the ASS treatment-controls rats most of the time stayed close to each other.

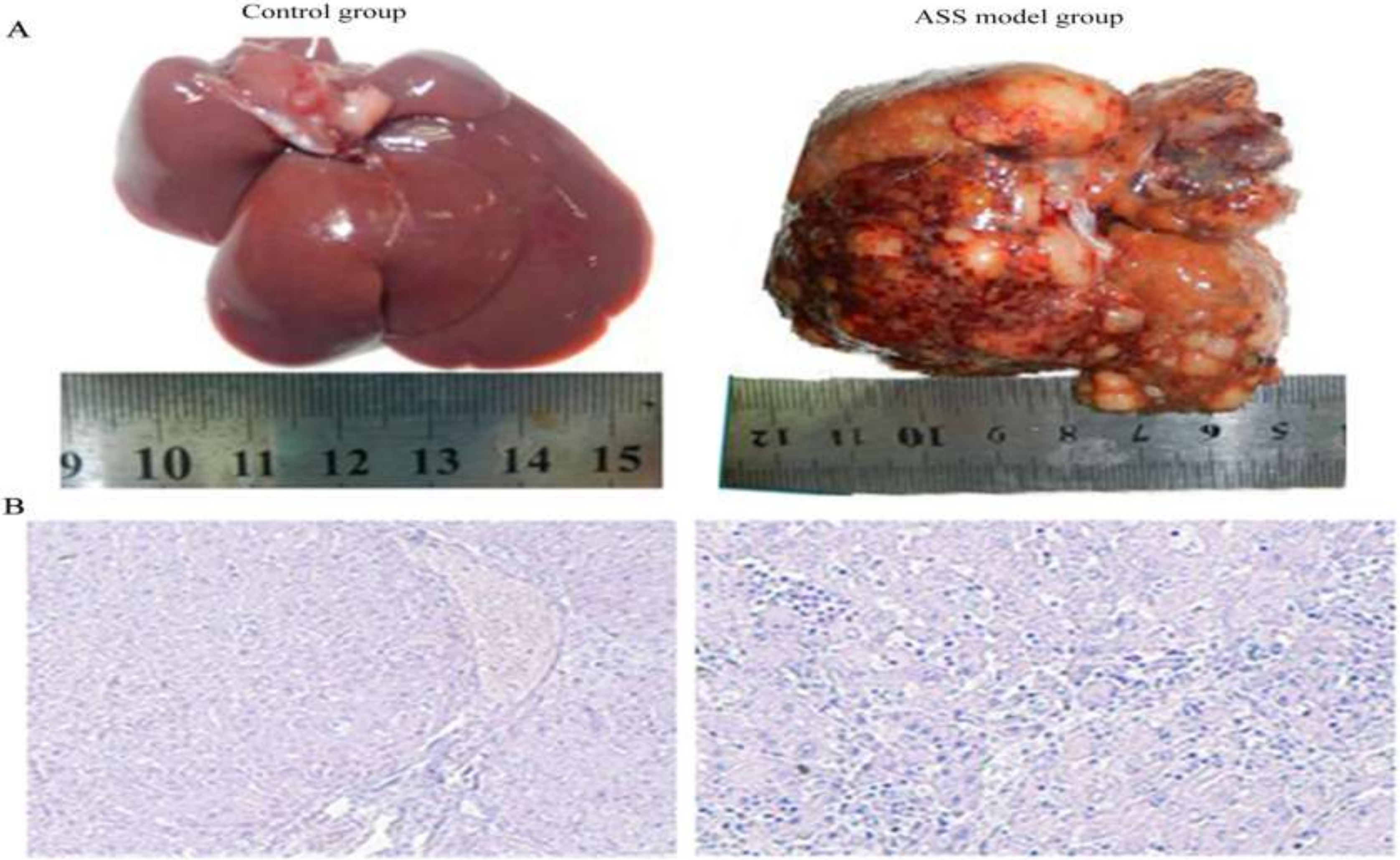

Figure S3. The hepatic general morphology and pathological changes at the 20th week

（A) The rat livers of the control group appeared normal, whereas the rat liver surface of the ASS treatment control group was rough and appeared pathological. （B) In the control rats the liver cells were arranged in order, and the sinus hepaticus was normal.There was no obvious edema or necrosis of hepatocytes. The liver cells showed granular degeneration, dark color, multiple sizes of grey white nodules and liver cell edema with obvious congestion and hepatocellular necrosis (piecemeal necrosis)and fibroplastic proliferation as well as form cell clusters (Magnification ×100).

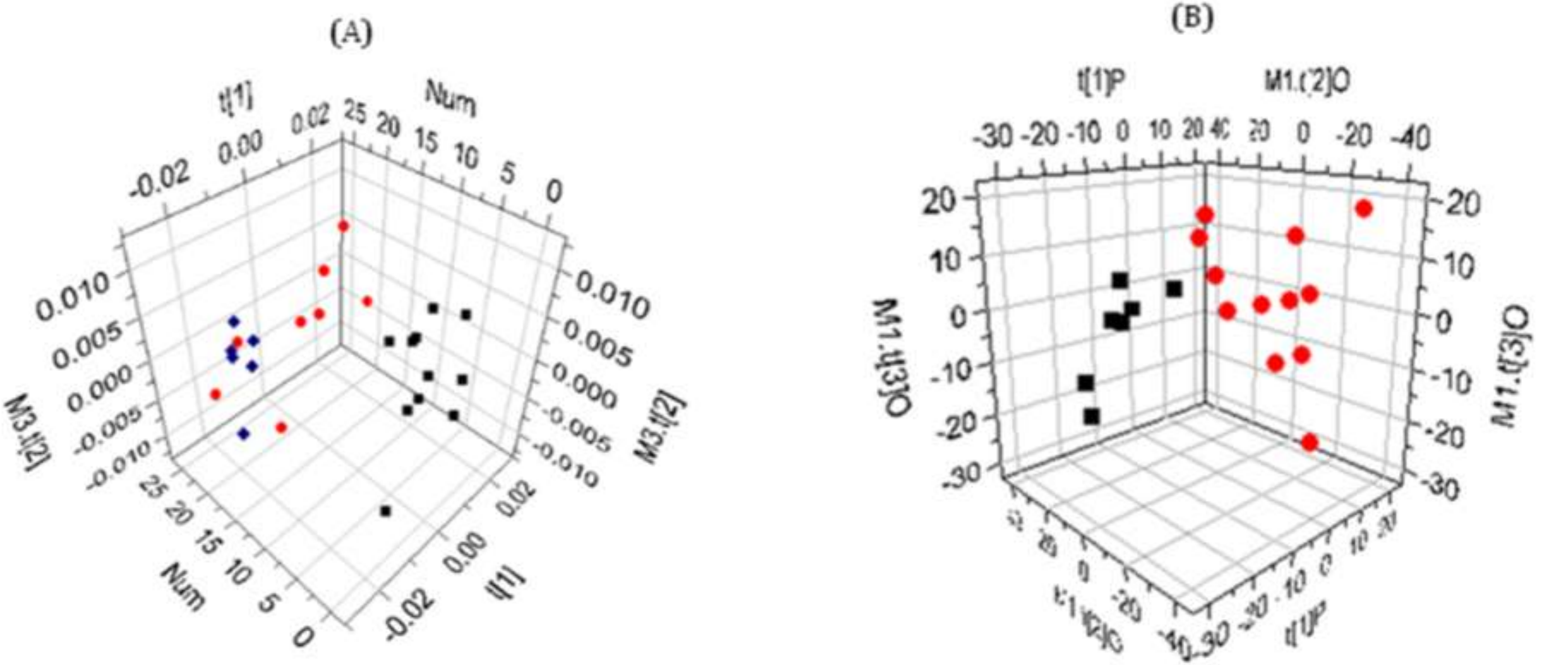

Notes: ■normal group; ●model group; ◆ASS model group Notes: ■ASS model group; ●ASMq medium dose group

Figure S4: 3D plots of PLS-DA scores in ^{1}H-NMR spectra.

A trend for unsupervised separation between these groups was found in the normal healthy group vs liver cancer control group vs ASS treatment control group (Figure S3A) as well as ASS treatment control group vs ASMq medium dose group (Figure S3B) score scatter plots. In order to get a better separation, OPLS-DA was applied in our study. Subsequently, the obtained parameters R^2X, which stands for the total explained variation of the model, and Q^2, which represents the predictability of the model, were used to describe the quality of these models. The coefficients indicating the significance of the metabolites contributing to the separation in serum are summarized in Tables 2A and 2B, respectively.

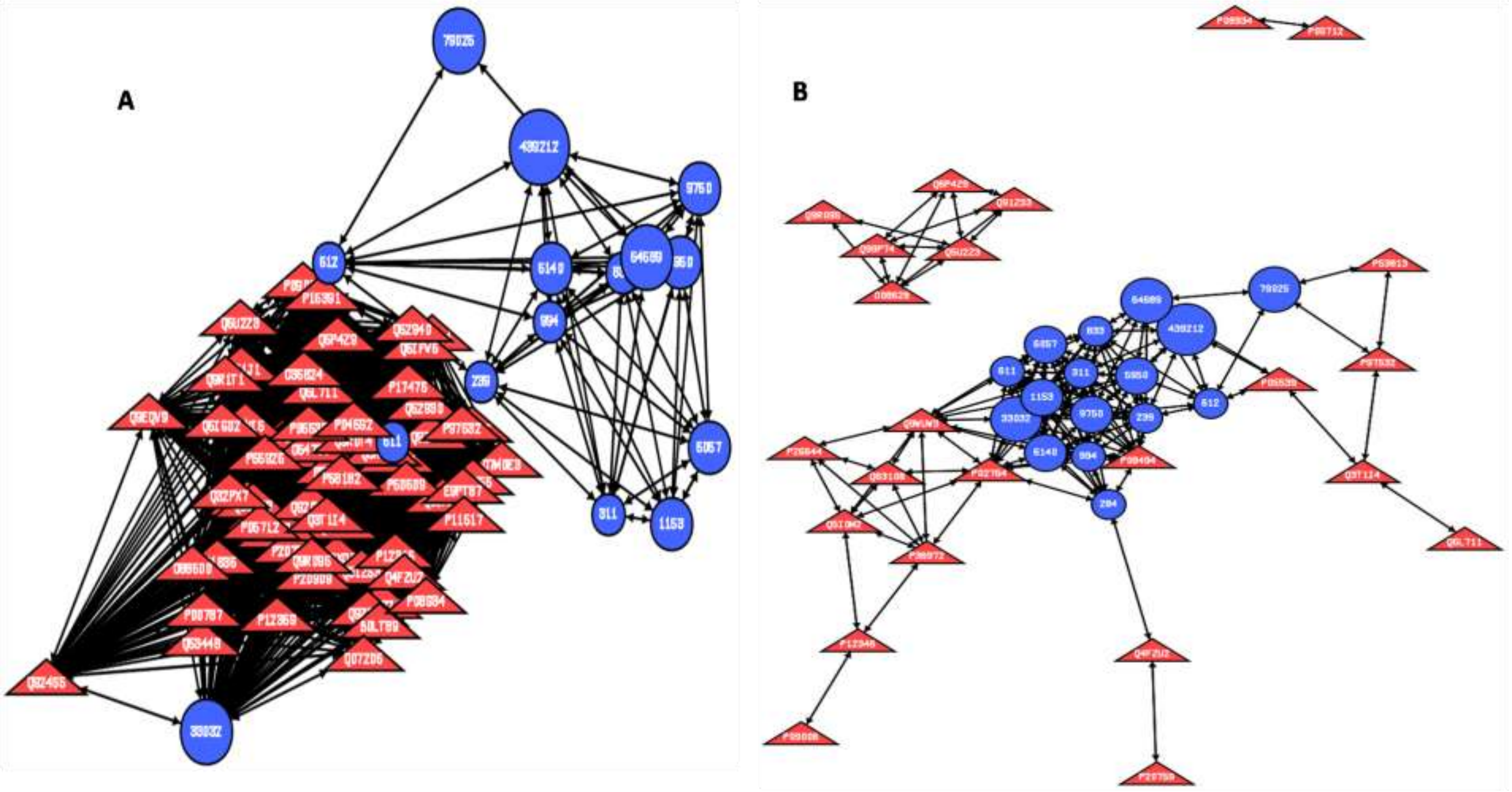

Figure S5: 3O mics-generated correlation network with proteomic and metabonomic analysis related to ASS HCC(A) and ASMq (B)groups.

Nodes denoted by triangles and circles represent proteins and metabolites, respectively. Enzyme and metabolite are presented in the context of the correlation network. The correlated relationships (PCC > 0.9) are represented by solid lines and the text-mining results between pairs of input molecules and literature-derived molecules are indicated by the dotted lines.
